# Supplementary material for: Universal scaling law for chiral antiferromagnetism
Source: Nat Commun. 2024 May 2;15:3717. doi: 10.1038/s41467-024-46325-5 (PMC11066068; doi:10.1038/s41467-024-46325-5)
Supplement: Supplementary file 1 — Supplementary Information [file 41467_2024_46325_MOESM1_ESM.pdf]

Supplementary Information for

Universal Scaling law for chiral antiferromagnetism

Shijie Xu[1,2,3,5+], Bingqian Dai[2+], Yuhao Jiang[1+], Danrong Xiong[1], Houyi Cheng[1,5], Lixuan Tai[2], Meng Tang[3], Yadong Sun[3], Yu He[1], Baolin Yang[4], Yong Peng[4], Kang L. Wang[2]* and Weisheng Zhao[1,5]*.

[+] Shijie xu, Bingqian Dai, and Yuhao Jiang contributed equally to this article

* Correspondence: klwang@ucla.edu, weisheng.zhao@buaa.edu.cn.

[1]Fert Beijing Institute, Ministry of Industry and Information Technology Key Laboratory of spintronics, School of Integrated Circuit Science and Engineering, Beihang University, 100191 Beijing, China

[2]Department of Electrical and Computer Engineering, University of California, Los Angeles, California 90095, United States

[3]Shanghai Key Laboratory of Special Artificial Microstructure, Pohl Institute of Solid State Physics and School of Physics Science and Engineering, Tongji University, Shanghai 200092, China

[4]Key Laboratory of Magnetism and Magnetic Materials of the Ministry of Education, School of Physical Science and Technology and Electron Microscopy Centre of Lanzhou University, Lanzhou University, Lanzhou 730000, P.R.China.

[5]Hefei Innovation Research Institute, Anhui High Reliability Chips Engineering Laboratory, Beihang University, Hefei 230013, China

1. Perpendicular magnetic anisotropy energy in the Mn_3Pt films
2. Linear scaling law of MnGe.
3. Linear scaling law in the Mn_3Sn, Mn_3Ir, Mn_3Ge films.
4. The measured Linear scaling law in the Mn_3Pt films.

Supplementary Note 1: Perpendicular magnetic anisotropy energy in the Mn_3Pt films

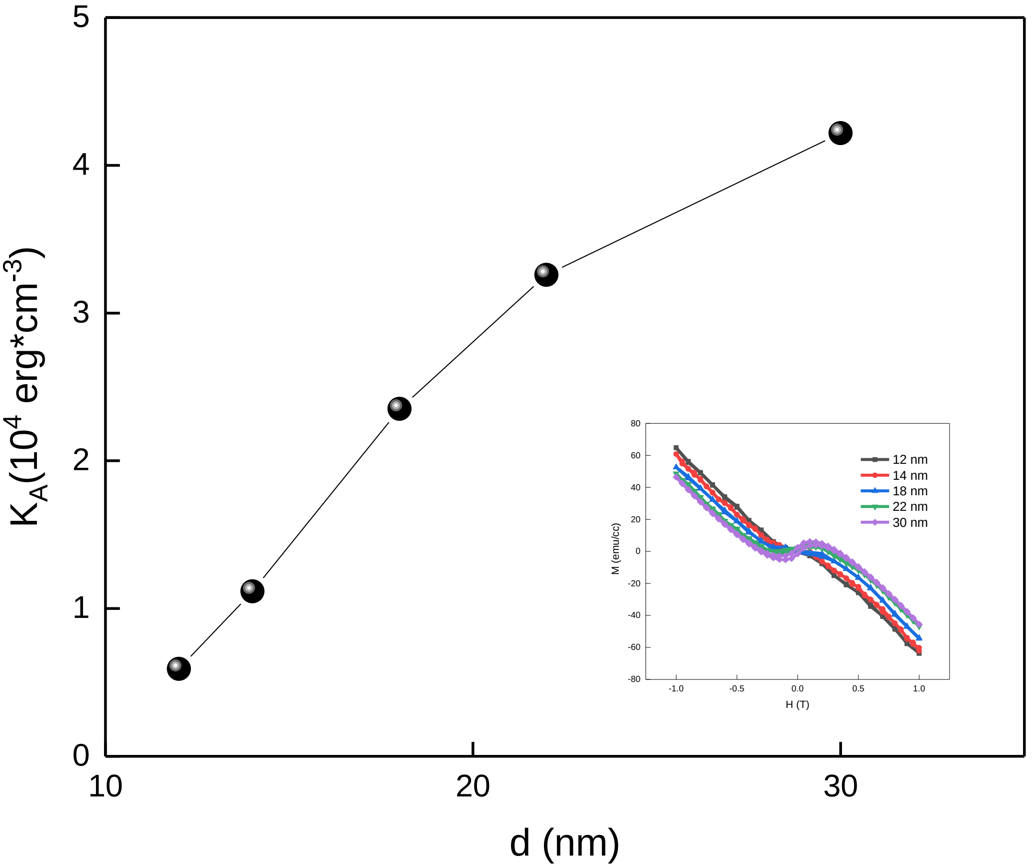

Supplementary Figure 1 | The measured perpendicular magnetic anisotropy energy K_A as a function of the thickness d in Mn_3Pt films. Inset is the M-H loop for Mn_3Pt films with different thickness at 300 K.

The antiferromagnetic magnetic anisotropy energy (due to spin canting effect) can be described as $K_A = |\int H_{\text{hard axis}} * dM - \int H_{\text{easy axis}} * dM|$. H is the magnetic field, M is the net moment. However, H. Chen et al. theoretically predicted the evolution of the magnetic structure about Mn_3Ir (which have the same structure of Mn_3Pt) [1]. The antiferromagnetic spin structure will tilt upward under positive saturation magnetic field, and have the opposite result under negative magnetic field. When the large magnetic field was applied at Hard axis, the net moment go to zero $\int H_{\text{hard axis}} * dM = 0$, So the $K_A = \int H_{\text{easy axis}} * dM$. Therefore, the perpendicular magnetic anisotropy energy in the Mn3Pt films with different thicknesses can described at figure 1. The magnetizations M induced by the net moment increases with the thickness, which means that the spin structure will tilt larger with the thickness. At a result, the scalar spin chirality will increase at larger thickness, causing the larger topological anomalous Hall effect.

Supplementary Note 2: Linear scaling law in the MnGe films.

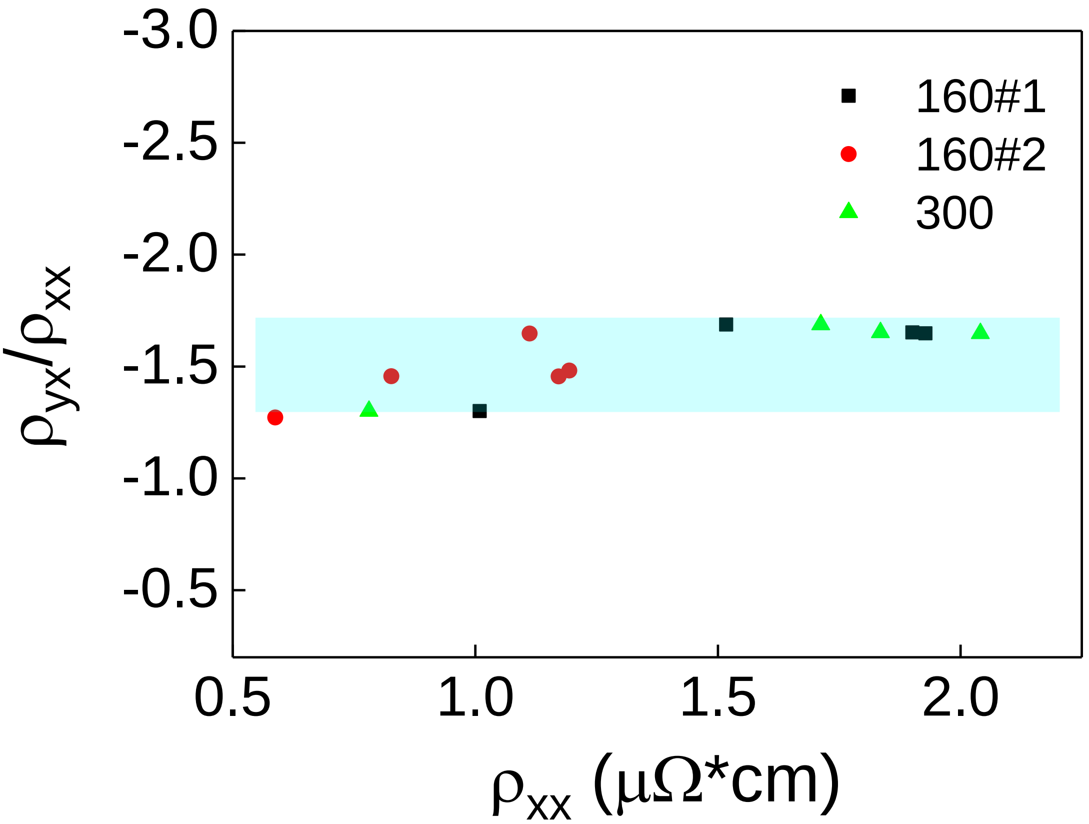

Supplementary Figure 2 | Universal scaling law for chiral magnets MnGe [2]. The various chiral AFM was plotted by $\frac{\rho_{AH}}{\rho_{xx}} = a_{sk} + b_{in}\rho_{xx}$. The slope and intercept represent the intrinsic and skew scattering anomalous Hall scaling factors, respectively.

The MnGe shows the linear (ρ_{xy} / ρ_{xx}) scaling relation for the anomalous Hall resistivity. In addition, the slope component is zero which means the skew-scattering in MnGe is dominated and the intrinsic contribution should be negligible. Both the 160 nm and 80 nm MnGe show the same linear scaling Law. One possibility for this unconventional skewscattering is the recently proposed "spin-chirality skew-scattering" mechanism [3], which caused by scalar spin chirality (SSC) or out-plane spin canting effect.

Supplementary Note 3: Linear scaling law in the Mn_3Sn, Mn_3Ir, Mn_3Ge films.

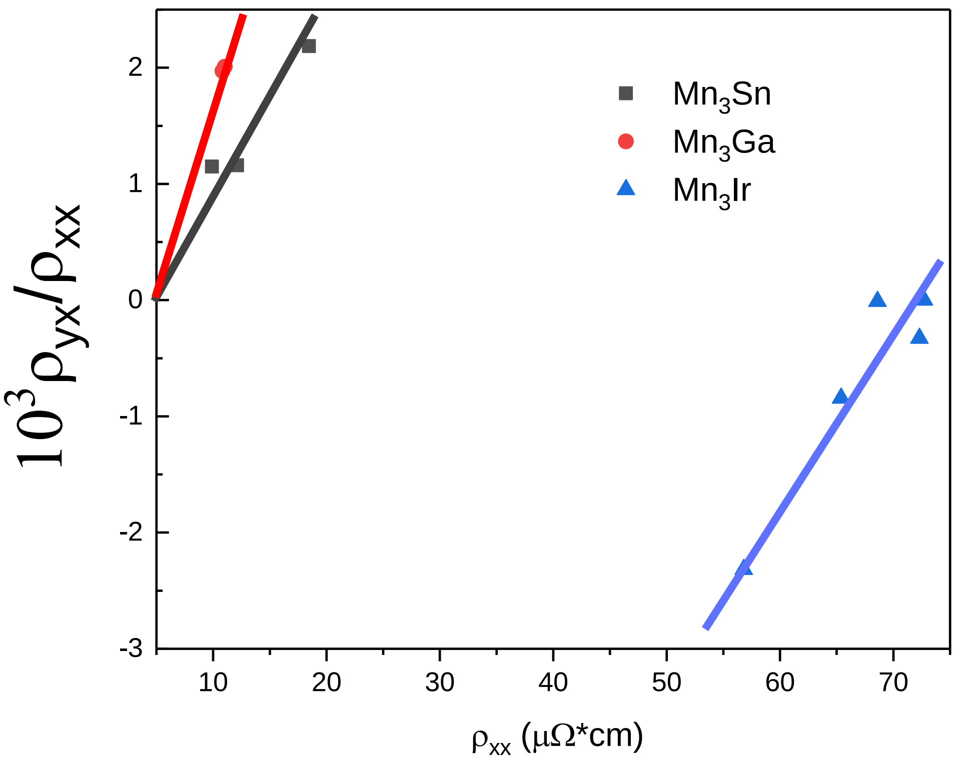

Supplementary Figure 3 | Universal scaling law for chiral magnets Mn_3Sn [5], Mn_3Ir [4], Mn_3Ge [5]. The various chiral AFM was plotted by $\frac{\rho_{AH}}{\rho_{xx}} = a_{sk} + b_{in}\rho_{xx}$.

The Mn_3Sn, Mn_3Ir and Mn_3Ge show the linear (ρ_{xy} / ρ_{xx}) scaling relation. For the Mn_3Sn and Mn_3Ge, the intercept component is zero which means the intrinsic mechanism is dominated and the skew scattering should be negligible. Because the Mn_3Sn and Mn_3Ge only have the in-plane spin canting effect (SSC=0) and the anomalous Hall effect come from intrinsic non-zero berry phase. For the Mn_3Ir film, both the intrinsic mechanism and skew scattering have non-negligible contribution.

Supplementary Note 4: The measured Linear scaling law in the Mn_3Pt films.

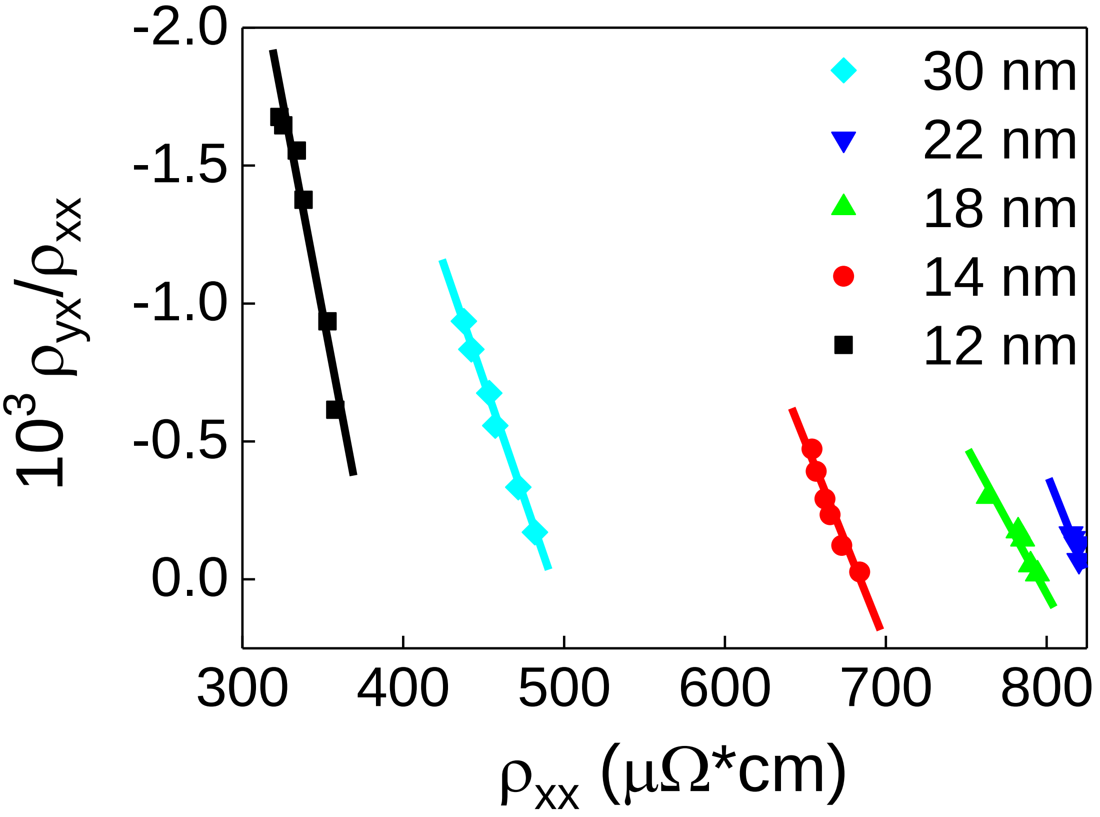

Supplementary Figure 4 | Universal scaling law for chiral magnets Mn_3Pt with the different thickness d. The various chiral AFM was plotted by $\frac{\rho_{AH}}{\rho_{xx}} = a_{sk} + b_{in}\rho_{xx}$.

Both the Mn_3Ir and Mn_3Pt have the same spin structure which have the large intrinsic anomalous Hall effect [1,6]. In addition, the corresponding spin texture at different fields is shown in the illustration figure (Fig. 1c, main text). The antiferromagnetic spin structure will tilt upward or downward under saturated magnetic field. As a sequence, the SSC will be be non-zero and induce a topological skew scattering AHE at the same time.

[1] Chen, H., Niu, Q. & MacDonald, A. H. Anomalous Hall effect arising from non-collinear antiferromagnetism.Phys. Rev. Lett. 112, 017205 (2014).
[2] Fujishiro, Y., Kanazawa, N., Kurihara, R., et al. Giant anomalous Hall effect from spin-chirality scattering in a chiral magnet[J]. Nature communications, 12(1): 1-6 (2021).
[3] Ishizuka, H. and Nagaosa, N. Spin chirality induced skew scattering and anomalous Hall effect in chiral magnets[J]. Science Advances, 4(2): eaap9962 (2018).
[4] Iwaki, H., Kimata, M., Ikebuchi, T. et al. Large anomalous Hall effect in L12-ordered antiferromagnetic Mn3Ir thin films [J]. Applied Physics Letters, 116(2): 022408 (2020).
[5] Chen, T., Tomita, T., Minami, S., et al. Anomalous transport due to Weyl fermions in the chiral antiferromagnets Mn3X, X= Sn, Ge[J]. Nature communications, 12(1): 1-14 (2021).
[6] Liu, Z. Q., Chen, H., Wang, J. M. et al. Electrical switching of the topological anomalous Hall effect in a non-collinear antiferromagnet above room temperature[J]. Nature Electronics, 1(3): 172-177(2018).
